# Supplementary material for: Mechanisms governing the pioneering and redistribution capabilities of the non-classical pioneer PU.1
Source: Nat Commun. 2020 Jan 21;11:402. doi: 10.1038/s41467-019-13960-2 (PMC6972792; doi:10.1038/s41467-019-13960-2)
Supplement: Supplementary file 7 — Source data [file 41467_2019_13960_MOESM7_ESM.zip › Source_Data/FigureS2/FigureS2B_MotifScanOutput/KmeansCluster1/homerResults/motif61.similar.html]

motif61

## Information for motif61

C
T
A
G
G
C
A
T
A
C
G
T
A
G
T
C
G
C
A
T
A
G
T
C
C
G
T
A
A
G
T
C
C
T
A
G
C
G
T
A
C
T
A
G
C
G
T
A
  
Reverse Opposite:  

C
G
A
T
G
A
T
C
G
C
A
T
A
G
T
C
T
C
A
G
A
C
G
T
T
C
A
G
C
G
T
A
A
C
T
G
G
T
C
A
C
G
T
A
G
A
T
C
  

|  |  |
| --- | --- |
| p-value: | 1e-14 |
| log p-value: | -3.274e+01 |
| Information Content per bp: | 1.778 |
| Number of Target Sequences with motif | 89.0 |
| Percentage of Target Sequences with motif | 1.75% |
| Number of Background Sequences with motif | 242.7 |
| Percentage of Background Sequences with motif | 0.61% |
| Average Position of motif in Targets | 109.3 +/- 45.6bp |
| Average Position of motif in Background | 106.0 +/- 84.2bp |
| Strand Bias (log2 ratio + to - strand density) | -0.3 |
| Multiplicity (# of sites on avg that occur together) | 1.01 |
| Motif File: | file (matrix) reverse opposite |

### Similar de novo motifs found

|  |  |  |  |  |  |  |  |
| --- | --- | --- | --- | --- | --- | --- | --- |
| Rank | Match Score | Redundant Motif | P-value | log P-value | % of Targets | % of Background | Motif file |
| 1 | 0.867 | A C T G C A G T A C G T A G T C A C G T A G T C C T G A A G C T C T A G C G T A | 1e-13 | -29.999186 | 1.73% | 0.64% | motif file (matrix) |
| 2 | 0.915 | C T A G G A C T A C G T T A G C G A C T T A G C T G C A G A T C T C A G C T G A T A C G | 1e-12 | -28.616677 | 1.30% | 0.41% | motif file (matrix) |
